# Exclusive Enteral Nutrition: Clinical Effects and Changes in Mucosal Cytokine Profile in Pediatric New Inflammatory Bowel Disease

**DOI:** 10.3390/nu11020414

**Published:** 2019-02-15

**Authors:** Helena Rolandsdotter, Kerstin Jönsson-Videsäter, Ulrika L. Fagerberg, Yigael Finkel, Michael Eberhardson

**Affiliations:** 1 Department of Clinical Science and Education, Södersjukhuset, Karolinska Institutet, 11883 Stockholm, Sweden; Yigael.finkel@sll.se; 2 Department of Gastroenterology, Sachs’ Children and Youth Hospital, Södersjukhuset, 11883 Stockholm, Sweden; 3 Clinical Immunology and Transfusion Medicine, Karolinska University Hospital, 14157 Huddinge, Sweden; kerstin.jonsson-videsater@sll.se; 4 Department of Medicine, Huddinge, Karolinska Institutet, 14157 Huddinge, Sweden; 5 Department of Women’s and Children’s Health, Karolinska Institutet, 17177 Stockholm, Sweden; ulrika.fagerberg@ki.se; 6 Center for Clinical Research, Västmanland Hospital, Uppsala University, 72189 Västerås, Sweden; 7 Department of Gastroenterology, Karolinska University Hospital, 17177 Stockholm, Sweden; Michael.eberhardson@ki.se; 8 Department of Medicine Solna, Karolinska Institutet, 17176 Stockholm, Sweden

**Keywords:** exclusive enteral nutrition, children, IBD, remission, mucosal cytokines

## Abstract

Exclusive Enteral Nutrition (EEN) is the first-line treatment in children with Crohn’s disease (CD) for induction of remission. However, the mode of action remains conjectural. The aim of this study was to investigate whether the effect of EEN is paralleled by changes in the mucosal cytokine profiles (MCP). Twelve children with new onset inflammatory bowel disease (IBD) received induction treatment with a polymeric EEN. We assessed clinical, endoscopic and histologic scoring before and after EEN. Twelve colonic cytokines were analyzed by Polymerase Chain Reaction (PCR) in six of the IBD patients at onset and after EEN as well as in six non-IBD control children at the diagnostic colonoscopy. Twelve children completed 6 weeks of EEN, except from one child who completed 4 weeks. At the control colonoscopy, 83% were in complete clinical remission. Changes were found in the MCPs of individual patients after EEN. In particular, children with IBD showed significantly higher values of Interleukin (IL)-12β (*p* = 0.008) and IL-23α (*p* = 0.02) compared to non-IBD controls at the diagnostic colonoscopy. Furthermore, an overall change in proinflammatory cytokines was noted in the IBD-group after treatment. Further studies are warranted to understand the role of EEN in MCP.

## 1. Introduction

Crohn’s disease (CD) and Ulcerative Colitis (UC), the major entities of pediatric IBD, are chronic inflammatory conditions of the gastrointestinal tract. Childhood IBD is characterized by more severe symptoms and wider disease extension at diagnosis compared to adults [1]. Abdominal pain, nausea and bloody diarrhea are common symptoms in both children and adults while growth retardation is frequently seen in pediatric patients [2]. EEN is the first-line treatment for remission induction of CD in pediatric patients. The clinical remission rate is 73–80% and it effectively induces mucosal healing (MH) at a rate that is superior to corticosteroids [3,4,5,6,7]. EEN consists of high energy liquid formulas, which are either elemental, semi-elemental or polymeric based. EEN is recommended to all children with luminal disease, including those with colonic involvement [3].

The immunological effects driven by EEN that contribute to mucosal healing (MH) are not yet fully understood [7]. The intestine hosts a majority of the body’s immune cells (70–80%) [8] and the interactions between a disrupted microbial composition, an impaired intestinal mucosal barrier and the mucosal immune system are considered to play an important role in the IBD development and its chronicity [7]. Some studies suggest that a deficiency of host immunity also contributes to the pathogenesis of IBD [9]. The cytokines, small immune-regulating messenger proteins, play a crucial part in the inflammatory response by regulating cell differentiation, producing proinflammatory cytokines and recruiting white blood cells from the bone marrow [10]. There are only a few studies that show that EEN seems to affect the expression of cytokines in the mucosa [11,12,13].

The aim of this study was to investigate the clinical effect of EEN as induction therapy and whether the effect of EEN is paralleled by changes in the mucosal cytokine profiles (MCP).

## 2. Materials and Methods

We conducted a prospective cohort study of children with new onset symptoms of possible IBD between August 2013 and September 2016. The inclusion criterion was previously healthy children up to 18 years of age with signs of IBD and the exclusion criterion was any use of immunosuppressive drugs within 6 months prior to inclusion. Thirteen children who fulfilled the ECCO/ESPGHAN consensus criteria for pediatric CD (14) entered the EEN study but one patient immediately left the study after the diagnostic endoscopy. One child received concomitant medication with mesalazine (53 mg/kg/day). Six patients who did not receive any IBD diagnosis were included as non-IBD controls. Demographic data are presented in Table 1.

### 2.1. Examinations and Tests

The patients were investigated according to the ECCO/ESPGHAN guidelines for pediatric IBD with initial laboratory investigations: peripheral blood count, liver enzymes, albumin, erythrocyte sedimentation rate (ESR), iron status, C-reactive protein (CRP), fecal calprotectin and stool cultures to exclude infectious etiology. In line with the guidelines, all patients were investigated with upper endoscopy and ileocolonoscopy under general anesthesia with multiple mucosal biopsies for histopathological evaluation [14]. For the study, four mucosal biopsies were obtained from the most inflamed site in the ileum or colon. The endoscopies were documented by a still picture system integrated in the computerized medical record program (Picsara, Mawell, Solna, Sweden). A control upper and lower endoscopy with biopsy harvesting at the same site as the diagnostic endoscopy was performed shortly after completion of EEN remission treatment in children with CD. No CC was performed in the six non-IBD controls. The body weight of study patients was obtained before and after the EEN treatment.

### 2.2. Clinical Assessment of Disease Activity and Disease Extension

We used the validated disease activity scoring PCDAI (Pediatric CD Activity Index) with <10 points for remission, 10–27.5 for mild disease, >27.5–37.5 moderate disease and >37.5–100 for severe disease. A PCDAI decrease of >12.5 points was regarded as a clinically significant response to treatment [15]. PCDAI score was assessed at inclusion and at CC. Disease extension was assessed by Paris classification [16].

### 2.3. Endoscopic Scoring

Simple Endoscopic Score for Crohn’s disease (SES-CD) was used to assess the mucosal inflammation in the CD patients [17] and was evaluated either by the endoscopist or by the investigator from the charts (the endoscopy history and endoscopy imaging). SES-CD describes the presence and size of ulcers, extent of the ulcerated surface, extent of the affected surface and the presence and type of narrowing scored with 0–3 points in rectum, sigmoid and left colon, transverse colon, right colon and ileum. Remission is equal to 0–2 points, mild disease to 3–6 points, moderate to 7–15 points and severe disease to ≥16 points.

### 2.4. Histopathology Scoring

The mucosal biopsies were reviewed by the validated histological Geboes scoring [18]. Grades < 3 were regarded as the absence of IBD inflammation, while grades ≥3 were regarded as the presence of IBD inflammation of varying degrees (3:1–3:7) with the following subgroups: a = basal plasmacytosis, b = lymphoid nodules, c = Paneth cell hyperplasia, d = eosinophils in the lamina propria, e = histiocytic cell proliferation, f = giant cells, g = granuloma, h = vasculitis and i = dysplasia.

### 2.5. Exclusive Enteral Nutrition (EEN)

Fortimel Energy^®^, a polymeric formula, was prescribed to meet a daily energy intake estimated at 120% of recommended daily allowance (RDA). Fortimel Energy^®^ contains 625 kJ, 5.8 g of fat and 5.9 g of protein/100 mL (Nutricia Advanced Medical Nutrition, Solna, Sweden). The EEN treatment was introduced by a daily step-wise increase by 25% of full daily intake for three days, which was followed by six weeks of EEN and rounded off by a step-wise decrease by 25% of full daily intake for 3 days. The children were allowed to drink clear beverages, eat a few popsicles and a few hard mints per day during the EEN treatment. Patients were allowed additional formula at their own discretion for daily satiation. At de-escalation, the patients were advised to avoid large amounts of dairy products, spicy food and large amount of legumes. The children and caregivers had one introductory session with a dietitian. Thereafter, the dietitian had weekly phone calls to monitor intake and motivate the patients and caregivers to complete the whole EEN treatment period. Scheduled clinical assessments were arranged after two weeks of EEN.

### 2.6. Cytokine-Selection

A panel of cytokines that are known to participate in chronic intestinal inflammation were selected for testing. We investigated Colony Stimulating Factor(CSF)-2, Interferon(IFN)-γ, Tumor Necrosis Factor(TNF)-α, Interleukin(IL)-1β, IL-4, IL-6, IL-10, IL-12β, IL-22, IL-23α, IL-36γ, Tumor Growth factor(TGF)-β1 and a control gene ABL. The amount of messenger-RNA (m-RNA) in the different proinflammatory mediators was compared to the signal from the ABL-gene and the result is presented as the cytokine/ABL ratio. We used TaqMan^®^ Gene Expression Assays, applied biosystems (Thermo Fischer Scientific^®^, Waltham, MA, USA)

### 2.7. RNA Extraction and Gene Expression by Quantitative Real-Time PCR

Biopsies for MCP were put into the RNA-later (Invitrogen, Waltham, MA USA) and kept at +6 °C for 24 h and then frozen (−20 °C) until analysis. Total RNA was isolated by utilizing the Fibrous tissue kit (Qiagen, Hilden, Germany) with slight modification. Defrosted and minced biopsies (3 mm) were homogenized with a pestle motor (WVR^®^, Radnor, PA, USA) for 30–60 s in 350 µL of RLT-buffer and 350 µL of 70% ethanol was added. The homogenates were loaded into spin columns and centrifuged before the columns were washed with 350 µL of RW1-buffer. Samples in the columns were treated by DNase I and washed with 350 µL of RW1-buffer, with the remainder handled according to the manufacturer protocol. cDNA was obtained by reverse transcription. Quantitative real-time PCR (qPCR) was performed using the 7500 Fast Real Time PCR System (Applied Biosystems, Foster City, CA, USA) for quantification. Probes were obtained from Applied Biosystems, TaqMan^®^ MGB probes, FAM™ dye-labeled: IL-1β (Hs00174097_m1), IL-4 (Hs00174122_m1), IL-6 (Hs00985639_m1), IL-10 (Hs00961622_m1), IL-12β (Hs001011518_m1), IL-22 (Hs01574154_m1), IL-23 (Hs00900828_g1), IL-36γ (Hs00219742_m1), TGF-β1 (Hs00998133_m1), TNF-α (Hs01113624_g1), IFN-γ (Hs00989291_m1), GM-CSF-2 (Hs00929873_m1) and ABL1 (Hs01104728_m1) according to the manufacturer protocol. Fold increases of mRNA transcripts were calculated as follows: ΔCt = Ct (gene of interest) − Ct (ABL1), ΔΔCt = ΔCt sample − average ΔCt control group and fold difference = 2−ΔΔCt. For technical reasons, CMCP was not analyzed in all children with IBD both at diagnosis and at CC. No active selection was performed.

### 2.8. Ethical Approval

The study was approved by the local Ethics Committee in Stockholm, Sweden (No. 2010/1252-31/1). Informed written consents were obtained from legal guardians for patients and also from all patients over 15 years of age before any study-related procedures were initiated in accordance with the Helsinki II Declaration.

### 2.9. Statistics

The comparisons between clinical chemistry values, SES-CD and disease activity index (PCDAI) before and after treatment were conducted with paired sample t-tests (parametric data) or Wilcoxon paired t-tests (non-parametric data) depending on the normal distribution (tested with Shapiro–Wilks test). Paired comparisons between cytokines before and after treatment were conducted with Wilcoxon paired t-tests while the comparisons between groups were conducted with Mann–Whitney U test after control of normality with Shapiro–Wilks test. All analyses were performed with the IBM SPSS Statistics Data Editor, version 23^®^ (Armonk, NY, USA). Statistical significance was set at *p* < 0.05.

## 3. Results

Thirteen children were included with a CD diagnosis according to the ECCO/ESPGHAN guidelines. One CD patient left the study after the first colonoscopy due to social circumstances. Out of 12 patients who completed EEN treatment, two patients were re-diagnosed with UC and one with IBD-unclassified (IBD-U) at the time of histopathological review after study completion (Table 1 for Paris classification at inclusion).

### 3.1. EEN Treatment

Eleven children completed six weeks of EEN and one CD patient completed only four weeks of EEN due to a lack of motivation, but fulfilled participation in all other aspects of the study. All patients accepted the prescribed EEN by oral intake without the use of nasogastric tube.

### 3.2. Clinical Activity Scoring

Clinical scoring of the children diagnosed with CD was measured by PCDAI, which was also used for the children who had their diagnoses changed to UC (*n* = 2) or IBD-U (*n* = 1). At inclusion, six children were considered to have mild disease (PCDAI 10–27.5) and seven patients showed moderate to severe disease (PCDAI > 27.5). There was a significant decrease in PCDAI at CC (*p* = 0.02). The median PCDAI was 26.5 (IQR 20.0–36.6, range 20–40) at inclusion and 5 (IQR 0-5, range 0–15) after EEN treatment. Ten of twelve patients (83%) showed clinical remission (PCDAI < 10) after EEN induction treatment.

### 3.3. Laboratory Values

ESR, CRP, hemoglobin, plasma-albumin and F-calprotectin were monitored in all patients. There were significant decreases in ESR (*p* = 0.005), CRP (*p* = 0.016) and f-calprotectin (*p* = 0.033) and significant increases in hemoglobin (*p* = 0.001) and p-albumin (*p* = 0.003) after EEN treatment (Table 2).

### 3.4. Anthropometric Data

There was a significant gain in weight (*p* = 0.003) after EEN treatment (Table 2).

### 3.5. Endoscopic Healing

All patients were examined with a control upper and lower endoscopy at a median of 13 days (range 1–45 days) after completion of EEN. A significant decrease (*p* = 0.008) was seen in SES-CD after EEN (median SES-CD 9.5, IQR 4.5–14.3, range 4–28 at inclusion compared to median 3.5, IQR 1–10, range 0–16 at CC).

### 3.6. Clinical Outcome of the Two UC Patients

One UC patient showed a reduction in PCDAI (23 to 5 points) and ESR (28 to 11 mm/h) and an increased hemoglobin value (110 to 116 g/L). However, no considerable effect was seen in SES-CD, CRP and F-calprotectin. The other UC patient showed a decrease in PCDAI (40 to 15 points), ESR (53 to 2 mm/h), CRP (47 to 11 mg/L), F-calprotectin (3169 to 1430 mg/kg) and SES-CD (21 to 16 points) and a raise in albumin (28 to 31 g/L) and Hb (83 to 128 g/L).

### 3.7. Mucosal Healing

The histopathological scoring using the Geboes score showed an improvement in ten patients, worsening in one patient and resolution of inflammation in one patient after EEN treatment (Table A1 and Table A2).

### 3.8. Adverse Events

Two patients reported initial mild abdominal pain related to the intake of nutritional drinks. The treatment was tolerated without any additional side effects in all 12 patients.

### 3.9. Mucosal Cytokine Profile

In six of twelve EEN-treated children, the cytokine profile in biopsies were analyzed both at diagnosis and at CC. In the remaining seven patients, mucosal cytokines in biopsies were analyzed either at diagnosis or at CC, but could not be analyzed both at diagnosis and CC due to technical circumstances.

#### 3.9.1. Cytokine Profiles in Six Patients Pre- and Post EEN

**Figure 1:**
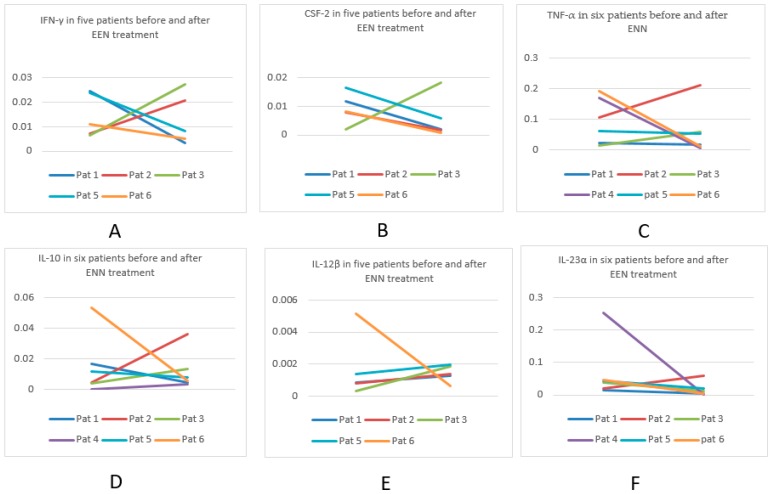
Six cytokine profiles in mucosal biopsies according to mRNA levels measured by q-PCR at disease onset and after EEN treatment. (**A**). IFN-γ at onset and after EEN treatment. (**B**). CSF-2 at onset and after EEN treatment. (**C**). TNF-α at onset and after EEN treatment. (**D**). IL-10 at onset and after EEN treatment. (**E**). IL-12β at onset and after EEN treatment. (**F**). IL-23α at onset and after EEN treatment.

No significant differences were found in any cytokine profile in six patients (five with CD and one with IBD-U) who completed six weeks of EEN. Furthermore, there were no associations in the cytokine profile in relation to gender, age or disease extension. In individual patients, both reductions and increases in the cytokine expression were seen. With regards to the key proinflammatory cytokine IL-12β, only one patient demonstrated a decrease while four patients showed an increase after EEN (one sample missing). In addition, the antiinflammatory IL-10 decreased in three patients and increased in three patients, while two patients had a reduction of the regulatory TGF-β1, one patient had no change and three patients showed increased expression after EEN treatment. The regulatory cytokine CSF-2 was downregulated in four out of five patients after EEN treatment (Table 3 and Figure 1). Table A3 for real-time PCR values per patient pre- and post-EEN.

#### 3.9.2. Cytokine Comparison between IBD Patients and Non-IBD Controls

When 12 MCPs in eight IBD-patients were compared to six non-IBD patients at inclusion, significantly higher values of IL-12β (*p* = 0.007) and IL-23α (*p* = 0.025) were measured in the IBD patients compared to non-IBD controls.

## 4. Discussion

In the present study, we aimed to investigate the effect of EEN treatment on symptoms, biochemistry, endoscopy, histopathology and mucosal cytokine profile in children with new onset IBD.

We found a significant reduction of PCDAI, CRP, ESR and F-calprotectin; a significant increase in hemoglobin and albumin levels; and a significant gain in weight between the baseline and after completion of the EEN treatment. Mucosal healing (MH), measured by SES-CD, was seen in the majority of the patients at CC compared to diagnosis. Our results are consistent with other reports, which have showed that EEN is effective for clinical and biochemical remission in up to 80% in children with newly diagnosed CD [19,20].

Interestingly, in the two patients with a subsequent change of diagnosis from CD to UC, EEN also had an antiinflammatory effect. To the best of our knowledge, the literature on the evaluation of EEN in UC is limited [21,22].

### MCP

In this pilot study, no significant differences in MCP were found after EEN treatment in children with IBD. However, we found significant higher levels of baseline IL-12β and IL-23α in the IBD-patients compared to non-IBD controls. These cytokines are known to play an important role in the pathogenesis of CD [23].

Our intention was to investigate the effects of EEN on the mucosal cytokine profile in addition to its clinical effects. It is likely that MCP mirrors the inflammatory activity more adequately than cytokines analyzed in peripheral blood [24]. Even though the reduced expression of proinflammatory cytokines was seen after EEN treatment, no consistent effect of EEN variations was identified. This is probably due to individual variations and a limited number of patients, which is an important limitation of the study.

The expression of TGF-β1, CSF-2 and IL-10, which are known as regulatory cytokines, demonstrated conflicting changes in the patient group after treatment. There are only a few reports published on mucosal cytokines after EEN. Yamamoto et al. investigated IL-1β, IL-1 receptor antagonist (IL-1ra), IL-6, IL-8 and TNF-α with enzyme-linked immunosorbent assay (ELISA) before and after four weeks of elemental diet in 28 adult patients. After treatment, a reduction in the mucosal expression of IL-1β, IL-1ra, IL-6, IL-8 and TNF-α was noticed. Furthermore, when they compared IBD patients to healthy controls, significantly higher levels of all cytokines were observed in the IBD patients [12].

Fell et al. investigated clinical efficacy and mucosal healing after eight weeks of CT3211 (a polymeric nutritional diet) in 29 children with active CD. In addition, IL-1β, IL-8, IL-10, TGF-β1 and IFN-γ were analyzed with PCR in ileal- and colonic biopsies from 18 children [11]. Complete clinical remission was seen in 79% of the patients. Furthermore, macroscopic and histological healing was associated with a significant downregulation of mucosal proinflammatory cytokines in ileum or/and colon. Elevated mucosal cytokines were observed in CD patients before EEN treatment compared to non-IBD controls and ileal TGF-β1 increased during treatment.

Thus, the reports on MCP and EEN show contradictory results and one explanation may be the diversity in methodology, but also in the types and length of EEN treatment. The individual expression of the mucosal cytokines may also reflect the diversity in patients and their immunological status since CD probably comprises several pathogenic mechanisms that differ between patients [25,26]. Nevertheless, earlier reports show aberrant gene expression in patients with CD, which involves cytokine expression. In 2006, Carr et al. found a strong association between an aberrant gene-expression of the proinflammatory cytokine IL23R and CD while Waschke et al. connected various single nucleotide polymorphisms in the TNF receptors to different CD phenotypes [27,28,29]. In our report, the expression of regulatory IL-10, TGF-β1 and CSF-2 was shown to decrease and increase in individual patients after EEN treatment. This may reflect the temporal aspect, i.e., the same cytokine may be protective during acute inflammation but harmful and perpetuate the chronic inflammation [30]. Thus, cytokines may exert variable functions during different immunological conditions [31,32,33].

## 5. Conclusions

Complete remission after 6 weeks of EEN was achieved in a majority of children with new onset IBD. We found a higher level of expression of IL-12β and IL-23α in these children compared to controls at inclusion. There were individual changes in the mucosal cytokine profiles after EEN-treatment but there were no consistent trends. Further understanding of the role of both antiinflammatory and regulatory cytokines as well as the aspects of temporal changes in the cytokine expression from diagnosis to remission requires larger studies.

## Figures and Tables

**Table 1:** Demography and Paris classification of 13 IBD patients at inclusion.

Sex (Girls/Boys)	6/7
Age in years, median (IQR) range	12.5 (10.5–14.5) 7.8–16.4
Symptom duration in months, median (IQR) range	6 (3.1–11.1) 1.5–12
Paris classification	No (=13)
A1aL1B1G1	1
A1bL1B1G0	4
A1bL2B1G0	4
A1bL2L4bB3G1	1
A1bL3L4aB1G0	1
A1bL2L4aB1G0	1
A1bL3L4aB1G0p	1

**Table 2:** Laboratory values and weight in 12 patients at inclusion and at control colonoscopy.

Value	At Inclusion Median (IQR) Range	After EEN Treatment Median (IQR) Range
Weight (kg)	37.9 (31.5–52.6) 27.5–59.7	40.5 (32.8–55.4) 29.8–59.1
ESR (mm/h)	28 (17.3–27.5) 8–53	9 (8–11) 2–21
CRP (mg/L)	14 (2–49.3) 1–86	2.5 (1–8.3) 1–20
Albumin (g/L)	33 (25–36) 24–41	36.3 (33.5–40.5) 31–44
Hemoglobin (g/L)	113 (101–119.5) 83–142	129 (121–135) 116–144
F-calprotectin (mg/kg)	2640 (1191–3169) 580–6360	620 (198–1556) 5–2256

**Table 3:** MCP in biopsies from six children at IBD onset and after treatment harvested from colon (in some patients, it was not possible to analyze all MCPs).

CYTOKINE	Decreased	Unchanged	Increased
CSF-2	4		1
IFN-γ	3		2
TNF-α	4		2
IL-1β	4		2
IL-4	3		
IL-6	2	2	2
IL-10	3		3
IL-12β	1		4
IL-22	2		
IL-23α	5		1
IL-36γ	4		1
TGF-β1	2	1	3

## Appendix A

**Table A1:** Geboes scoring: Grades < 3 were regarded as absence of IBD inflammation, while grades ≥ 3 were regarded as presence of IBD inflammation of varying degree (3:1–3:7) with the following subgroups: a = basal plasmacytosis, b = lymphoid nodules, c = Paneth cell hyperplasia, d = eosinophils in the lamina propria, e = histiocytic cell proliferation, f = giant cells, g = granuloma, h = vasculitis and i = dysplasia. The table shows 11 segments (segment 1 = ileum, segment 11 = rectum) before and after EEN treatment.

	Segm. 1	Segm. 2	Segm. 3	Segm. 4	Segm. 5	Segm. 6	Segm. 7	Segm. 8	Segm. 9	Segm. 10	Segm. 11
Patient Endoscopy1|Endoscopy2											
Pat 1 CD	mult.g|g	<3|<3	<3|<3	<3|<3	<3|<3	<3|<3	<3|<3	<3|<3	<3|<3	<3|<3	<3|<3
Pat 2 UC	<3| 3:3	5|3:7	3:7|3:7	3:3|3:3	3:3|3:7	5|3:7	5|3:3	4|3:7	3:7|3:7		<3|3:7
Pat 3 CD	<3|<3	<3+g|<3	5+b|<3+e	<3|mult.g	<3|<3+e	<3|<3+e	<3|<3	<3|<3+g	<3|<3	<3|<3	<3|<3
Pat 4 CD	3:3+b+g|3:1	3:3+b+g|3:1	3:3+b+g|3:1	<3|<3	<3|<3	<3|<3	<3|<3	3:3|<3	<3|<3	<3|<3	<3|<3
Pat 5 CD	<3|<3	<3|<3	<3|<3	<3|<3	<3|<3	<3|<3	<3|<3	<3|<3	<3|<3	<3|<3	<3|<3
Pat 6 CD	3:3+e|<3	<3|<3	<3|<3	<3|<3	<3|<3	<3|<3	<3|<3	<3|<3	<3|<3	<3|<3	4+g|<3
Pat 7 UC	<3|<3	3:7|3:3	3:7+e|3:3	3:7|3:3	3:7|3:3	3:3|3:3	3:7|3:7	3:7|3:3	4+e|3:7	3:7+c|3:3	3:7|3:3
Pat 8 CD	infl.polyp|3:7	<3|<3+g	<3+g|<3	<3+e|3:3	<3+g|3:3	3:2|<3	3:2+g|3:1	<3|<3	<3+g|<3	<3+g|<3	<3+g|<3
Pat 9 I-BDU	<3|<3	<3|<3	<3|<3	3:2|<3	<3|<3	<3|<3	3:1|<3	3:1|<3	3:1|<3	<3|<3	<3|<3
Pat 10 CD	<3+g|<3+g	<3+g|<3+g	<3|<3+g	<3|<3	<3+g|<3	<3|<3	<3|<3+g	<3+g|<3	<3|<3	<3+g|<3	<3+g|<3
Pat 11	<3|<3	<3|<3	3:4|<3	3:7+e|<3	3:7+e|<3	4+e|<3	4|<3:4	4|<3	4|3:4	3:7+e|<3	4|<3
Pat 12	5+e|3:2	3:3|3:2	3:3|3:2	<3|<3	<3|3:7	<3|3:7	<3|<3	<3|<3	<3|<3	<3|<3	3:7|<3

**Table A2:** Geboes results summarized (every patient, 11 segments) as improvement, no change, aggravated inflammation or no inflammation after EEN.

Patient	Improvement	No Change	Aggravated Inflammation	No Inflammation
1	1			10
2	4	4	3	
3	2		4	5
4	4			7
5				11
6	2	1		8
7	8		3	
8	7	2	1	1
9	4			7
10	4	5	2	
11	9			2
12	4		2	5

**Table A3:** Real-time PCR values per patient pre- and post-EEN and mean values and SD for group per cytokine.

**Patient**	**CSF-2** **RQ pre-post EEN**	**IFN-γ** **RQ pre-post EEN**	**TNF-α** **RQ pre-post EEN**	**IL1-β:1** **RQ pre-post EEN**	**IL-4:1** **RQ pre-post EEN**	**IL-6:1** **RQ pre-post EEN**
1	0.011651–0.001997	0.024588–0.003389	0.024577–0.016915	0.573893–0.020431	0.000275–4.72 × 10^–5^	0.001001–0.001825
2	0.007851–0.001629	0.007228–0.020651	0.106451–0.21017	0.051899–0.924653	0.001325–########	0.020791–0.172768
3	0.001862–0.018126	0.00664–0.02729	0.014612–0.059869	0.218129–0.408354	0.000282–8.68 × 10^–5^	0.008237–0.029142
4	########–########	########–0.00048	0.168562–0.007131	1.021516–0.010524	########–########	########–0.003238
5	0.016589–0.005784	0.023729–0.008329	0.06296–0.054617	2.175898–0.061581	0.000737–0.000148	0.042764–0.022326
6	0.008264–0.000895	0.010918–0.00516	0.190799–0.012544	3.225579–0.108797	########–########	0.32355–0.00282
Mean for group pre-post EEN	0.0092434–0.005686	0.014621–0.010883	0.09466–0.060208	1.211152–0.255723	0.0006548–9.39959 × 10^−5^	0.158537–0.038687
SD for group pre-post EEN	0.0048404–0.006446	0.007929–0.009732	0.09466–0.060208	1.137297–0.328006	0.0004296–4.14339 × 10^−5^	0.122961–0.06088
**Patient**	**IL–10:1** **RQ pre–post EEN**	**IL–12β:1** **RQ pre–post EEN**	**IL–22:1** **RQ pre–post EEN**	**IL–23α:1** **RQ pre–post EEN**	**IL–36γ:1** **RQ pre–post EEN**	**TGFβ1:1** **RQ pre–post EEN**
1	0.016987–0.004407	0.000851–0.001291	#######–0.002077	0.015346–0.005568	0.000435–0.000911	0.653298–0.657096
2	0.004295–0.03603	0.000767–0.001354	########–0.016029	0.020307–0.060188	0.002836–0.002475	1.013262–4.188852
3	0.003799–0.013653	0.000296–0.001842	0.000458–0.004207	0.037239–0.012223	0.002853–0.002125	0.425452–0.726292
4	########–0.003523	########–########	########–#######	0.254504–0.003021	########–########	1.497149–0.271375
5	0.01185–0.007582	0.001391–0.001984	0.014448–0.008729	0.044249–0.019889	0.00407–0.000911	0.988215–1.261974
6	0.053329–0.005778	0.005143–0.000639	0.008147–8.46 × 10^−5^	0.046621–0.004837	0.001272–0.000563	0.83872–0.52586
Mean for group pre-post EEN	0.018052–0.011829	0.00169–0.001422	0.007684–0.006225	0.069711–0.017621	0.002293–0.001397	0.902683–1.271908
SD for group pre-post EEN	0.018311–0.011312	0.001761–0.000475	0.005721–0.005681	0.083447–0.019867	0.001285–0.000756	0.333408–1.338034

## References

[1] Van Limbergen J., Russell R.K., Drummond H.E., Aldhous M.C., Round N.K., Nimmo E.R., Smith L., Gillett P.M., McGrogan P., Weaver L.T. (2008). Definition of phenotypic characteristics of childhood-onset inflammatory bowel disease. Gastroenterology.

[2] Baumgart D.C., Sandborn W.J. (2012). Crohn’s disease. Lancet.

[3] Ruemmele F.M., Veres G., Kolho K.L., Griffiths A., Levine A., Escher J.C., Amil Dias J., Barabino A., Braegger C.P., Bronsky J. (2014). Consensus guidelines of ECCO/ESPGHAN on the medical management of pediatric Crohn’s disease. J. Crohns Colitis.

[4] Zachos M., Tondeur M., Griffiths A.M. (2008). Enteral nutritional therapy for induction of remission in Crohn’s disease. Cochrane Database Syst. Rev..

[5] Dziechciarz P., Horvath A., Shamir R., Szajewska H. (2007). Meta-analysis: Enteral nutrition in active Crohn’s disease in children. Aliment. Pharmacol. Ther..

[6] Day A.S., Whitten K.E., Lemberg D.A., Clarkson C., Vitug-Sales M., Jackson R., Bohane T.D. (2006). Exclusive enteral feeding as primary therapy for Crohn’s disease in Australian children and adolescents: A feasible and effective approach. J. Gastroenterol. Hepatol..

[7] Cuiv P.O., Begun J., Keely S., Lewindon P.J., Morrison M. (2016). Towards an integrated understanding of the therapeutic utility of exclusive enteral nutrition in the treatment of Crohn’s disease. Food Funct..

[8] Furness J.B., Kunze W.A., Clerc N. (1999). Nutrient tasting and signaling mechanisms in the gut. II. The intestine as a sensory organ: Neural, endocrine and immune responses. Am. J. Physiol..

[9] Korzenik J.R., Dieckgraefe B.K. (2000). Is Crohn’s disease an immunodeficiency? A hypothesis suggesting possible early events in the pathogenesis of Crohn’s disease. Dig. Dis. Sci..

[10] Neurath M.F. (2014). Cytokines in inflammatory bowel disease. Nat. Rev. Immunol..

[11] Fell J.M., Paintin M., Arnaud-Battandier F., Beattie R.M., Hollis A., Kitching P., Donnet-Hughes A., MacDonald T.T., Walker-Smith J.A. (2000). Mucosal healing and a fall in mucosal proinflammatory cytokine mRNA induced by a specific oral polymeric diet in paediatric Crohn’s disease. Aliment. Pharmacol. Ther..

[12] Yamamoto T., Nakahigashi M., Umegae S., Kitagawa T., Matsumoto K. (2005). Impact of elemental diet on mucosal inflammation in patients with active Crohn’s disease: Cytokine production and endoscopic and histological findings. Inflamm. Bowel Dis..

[13] Schwerd T., Frivolt K., Clavel T., Lagkouvardos I., Katona G., Mayr D., Uhlig H.H., Haller D., Koletzko S., Bufler P. (2016). Exclusive enteral nutrition in active pediatric Crohn disease: Effects on intestinal microbiota and immune regulation. J. Allergy Clin. Immunol..

[14] Levine A., Koletzko S., Turner D., Escher J.C., Cucchiara S., de Ridder L., Kolho K.L., Veres G., Russell R.K., Paerregaard A. (2014). ESPGHAN revised porto criteria for the diagnosis of inflammatory bowel disease in children and adolescents. J. Pediatr. Gastroenterol. Nutr..

[15] Turner D., Griffiths A.M., Walters T.D., Seah T., Markowitz J., Pfefferkorn M., Keljo D., Waxman J., Otley A., LeLeiko N.S. (2012). Mathematical weighting of the pediatric Crohn’s disease activity index (PCDAI) and comparison with its other short versions. Inflamm. Bowel Dis..

[16] Levine A., Griffiths A., Markowitz J., Wilson D.C., Turner D., Russell R.K., Fell J., Ruemmele F.M., Walters T., Sherlock M. (2011). Pediatric modification of the Montreal classification for inflammatory bowel disease: The Paris classification. Inflamm. Bowel Dis..

[17] Daperno M., D’Haens G., Van Assche G., Baert F., Bulois P., Maunoury V., Sostegni R., Rocca R., Pera A., Gevers A. (2004). Development and validation of a new, simplified endoscopic activity score for Crohn’s disease: The SES-CD. Gastrointest. Endosc..

[18] Geboes K., Riddell R., Ost A., Jensfelt B., Persson T., Lofberg R. (2000). A reproducible grading scale for histological assessment of inflammation in ulcerative colitis. Gut.

[19] Day A.S., Lopez R.N. (2015). Exclusive enteral nutrition in children with Crohn’s disease. World J. Gastroenterol..

[20] Bannerjee K., Camacho-Hubner C., Babinska K., Dryhurst K.M., Edwards R., Savage M.O., Sanderson I.R., Croft N.M. (2004). Antiinflammatory and growth-stimulating effects precede nutritional restitution during enteral feeding in Crohn disease. J. Pediatr. Gastroenterol. Nutr..

[21] Gonzalez-Huix F., Fernandez-Banares F., Esteve-Comas M., Abad-Lacruz A., Cabre E., Acero D., Sanderson I.R., Croft N.M. (1993). Enteral versus parenteral nutrition as adjunct therapy in acute ulcerative colitis. Am. J. Gastroenterol..

[22] Shaoul R., Brown S., Day A.S. (2018). Reasoning Beyond the Potential Use of Exclusive Enteral Nutrition and Other Specified Diets in Children with Ulcerative Colitis. J. Pediatr. Gastroenterol. Nutr..

[23] Guan Q., Zhang J. (2017). Recent Advances: The Imbalance of Cytokines in the Pathogenesis of Inflammatory Bowel Disease. Mediators Inflamm..

[24] Dock J., Ramirez C.M., Hultin L., Hausner M.A., Hultin P., Elliott J., Yang O.O., Anton P.A., Jamieson B.D., Effros R.B. (2017). Distinct aging profiles of CD8+ T cells in blood versus gastrointestinal mucosal compartments. PLoS ONE.

[25] Roggenbuck D., Reinhold D., Baumgart D.C., Schierack P., Conrad K., Laass M.W. (2016). Autoimmunity in Crohn’s Disease-A Putative Stratification Factor of the Clinical Phenotype. Adv. Clin. Chem..

[26] Cleynen I., Boucher G., Jostins L., Schumm L.P., Zeissig S., Ahmad T., Andersen V., Andrews J.M., Annese V., Brand S. (2016). Inherited determinants of Crohn’s disease and ulcerative colitis phenotypes: A genetic association study. Lancet.

[27] Ek W.E., D’Amato M., Halfvarson J. (2014). The history of genetics in inflammatory bowel disease. Ann. Gastroenterol..

[28] Duerr R.H., Taylor K.D., Brant S.R., Rioux J.D., Silverberg M.S., Daly M.J., Steinhart A.H., Abraham C., Regueiro M., Griffiths A. (2006). A genome-wide association study identifies IL23R as an inflammatory bowel disease gene. Science.

[29] Waschke K.A., Villani A.C., Vermeire S., Dufresne L., Chen T.C., Bitton A., Cohen A., Thomson A.B., Wild G.E. (2005). Tumor necrosis factor receptor gene polymorphisms in Crohn’s disease: Association with clinical phenotypes. Am. J. Gastroenterol..

[30] Bamias G., Cominelli F. (2016). Cytokines and intestinal inflammation. Curr. Opin. Gastroenterol..

[31] Sanjabi S., Zenewicz L.A., Kamanaka M., Flavell R.A. (2009). Antiinflammatory and proinflammatory roles of TGF-beta, IL-10 and IL-22 in immunity and autoimmunity. Curr. Opin. Pharmacol..

[32] Egea L., Hirata Y., Kagnoff M.F. (2010). GM-CSF: A role in immune and inflammatory reactions in the intestine. Expert Rev. Gastroenterol. Hepatol..

[33] Dabritz J. (2014). Granulocyte macrophage colony-stimulating factor and the intestinal innate immune cell homeostasis in Crohn’s disease. Am. J. Physiol. Gastrointest. Liver Physiol..

